# Memorial Window to Horace Wells

**Published:** 1903-05

**Authors:** 


### MEMORIAL WINDOW TO HORACE WELLS.

SUNDAY, April 12, witnessed an imposing service in Center Church, Hartford, Conn., in the placing of an artistic and symbolic window by Charles T. Wells, in memory of his father, the discoverer of anæsthesia, and of his mother, Elizabeth Wales Wells.

The Hartford Dental Society and the Hartford Medical Society attended as bodies, and were represented, the Dental Society by Dr. James McManus, its president, with some sixty members, and the Medical Society by Dr. William T. Bacon, president, and forty members; and in addition many visiting dentists and State and city officials.

"The 'Wells window,' as it will be called, is peculiarly rich in its deep and soft colorings, the figures are expressive, the posing effective, and the general plan in complete harmony with the windows which are near it. It is from the brush of Frederick Wilson, of the Tiffany studios in New York, and is of much artistic beauty. The subject of the large decorative panel of the window is symbolical of the great work accomplished by Dr. Horace Wells in his discovery of anæsthesia. A sitting female figure with helmet and armor, sword in hand, and a shield with which she protects a kneeling figure, represents Righteousness, Faith, and Salvation, while the kneeling figure, with a white dove clasped to her bosom and wearing a wreath, symbolizes Mercy and Peace. The two are artistically posed beneath an ornamental arch supported by decorative pillars, the arrangement of the draperies and the expression of the countenances being handled remarkably well. As the kneeling figure seems to be imbued with a feeling of security on account of the protecting influence of the other, so does mankind rest secure in the knowledge that, owing to the research of Dr. Wells, it has security from the sufferings it was once called upon to endure.

"The inscription in the upper part of the window, 'Mercy and Truth are together; Righteousness and Peace have kissed each other,' is from Psalms lxxxv. 10, and, in view of the general recognition of the work that Dr. Wells did, is in a way symbolic of the close of the contest over the discovery. On one of the lower panels is the text from Revelation xx. 4, 'Neither shall there be any more pain: for the former things are passed away,' beautifully symbolizing the great benefit Dr. Horace Wells was to mankind by the application of anæsthesia to surgery."

